# Anomaly Detections for Manufacturing Systems Based on Sensor Data—Insights into Two Challenging Real-World Production Settings

**DOI:** 10.3390/s19245370

**Published:** 2019-12-05

**Authors:** Klaus Kammerer, Burkhard Hoppenstedt, Rüdiger Pryss, Steffen Stökler, Johannes Allgaier, Manfred Reichert

**Affiliations:** 1 Institute of Databases and Information System, University of Ulm, 89081 Ulm, Germany; burkhard.hoppenstedt@uni-ulm.de (B.H.); manfred.reichert@uni-ulm.de (M.R.); 2 Institute of Clinical Epidemiology and Biometry, University of Würzburg, 97080 Würzburg, Germany; ruediger.pryss@uni-wuerzburg.de; 3 Uhlmann Pac-Systeme GmbH & Co. KG, 88471 Laupheim, Germany; stoekler.s@uhlmann.de; 4 ATR Software GmbH, 89231 Neu-Ulm, Germany; johannes.allgaier@uni-ulm.de

**Keywords:** anomaly detection, sensor data, machine learning, production machines

## Abstract

To build, run, and maintain reliable manufacturing machines, the condition of their components has to be continuously monitored. When following a fine-grained monitoring of these machines, challenges emerge pertaining to the (1) feeding procedure of large amounts of sensor data to downstream processing components and the (2) meaningful analysis of the produced data. Regarding the latter aspect, manifold purposes are addressed by practitioners and researchers. Two analyses of real-world datasets that were generated in production settings are discussed in this paper. More specifically, the analyses had the goals (1) to detect sensor data anomalies for further analyses of a pharma packaging scenario and (2) to predict unfavorable temperature values of a 3D printing machine environment. Based on the results of the analyses, it will be shown that a proper management of machines and their components in industrial manufacturing environments can be efficiently supported by the detection of anomalies. The latter shall help to support the technical evangelists of the production companies more properly.

## 1. Introduction

For manufacturing companies, the management of machine failures is becoming increasingly important. Due to the increasing complexity of the machines, downtimes of any kind can affect the overall success of a company. Buying a spare system is not an alternative solution as the acquisition costs normally surpass the benefit of the spare system. In addition, most replacement systems also require regular maintenance, even if they are not used. That is why companies are looking for new ways to manage machine failures cost-effectively. Predictive Maintenance is one topical subject, among others, which is a promising direction to tackle machine failures before they actually occur. However, the selection of appropriate techniques from the field of Predictive Maintenance is challenging as numerous aspects have to be considered [1]. In addition to Predictive Maintenance, there are many other approaches in this context for coping with machine failures such as Condition Monitoring or Continuous Improvements. Moreover, the trend towards machine learning raises the question of whether machine failures can be easily predicted. Although technical developments have improved the possibilities for manufacturing companies to cope with machine breakdowns, their practical application is still a challenging task for many reasons. On the basis of these considerations, the work at hand presents two real-world cases that were carried out in cooperation with manufacturing companies. For these companies, the detection of system errors is of utmost importance. In this context, it was shown that the detection of anomalies [2] of a machine is crucial for these companies. However, the meaningful detection of such anomalies is very complex. Interestingly, so far, many manufacturing companies often employ a selected choice of technical evangelists that are only able to detect anomalies based on their practical experiences over time. Such experts, in turn, are very expensive by design. To relieve them from manual decisions, this paper elaborates on how machine failures of these scenarios can be managed by analyzing sensor data of the production machines. In particular, the two examples will show that different types of sensor data, as well as detection techniques, should be considered. The first presented real-world setting is related to pharma packing machines. The latter machines wrap tablets into individual packaging units (i.e., blisters), and usually comprise several other components. For example, a product loader component pushes blisters and leaflets into cartons. This procedure, in turn, is prone to errors. Therefore, the packaging process needs to be continuously monitored to reduce costly downtimes as well as to comply with federal regulations. The continuous monitoring procedure, in turn, generates a large amount of sensor data coming from sensors that are related to the several components of the packaging machine. In addition, the pharma packing machine can be individualized for each customer, which might lead to many sensor parameter settings of the same machine type, including all obscured components.

The second presented real-world setting is related to 3D printing machines in the field of optical products. In contrast to the first example, in the second setting, not only sensor values from the machine itself are important, but also sensors that measure the environment of the machine. Note that this fact distinguishes this scenario from the first one. Here, for example, an increase in the environmental temperature or humidity may have a significant influence on the production process of the manufacturing machine. Anomalies, such as room temperature that has negative effects on the machines, should, therefore, be avoided.

Regarding the anomaly detection in general, plenty of algorithms were proposed that address different use cases [3]. The main objective of these algorithms is to analyze streaming data to develop models that can be used with an appropriate number of parameters and across applications, i.e., for machines with different characteristics and purposes. As in most practical cases, a very large number of sensor values must be analyzed simultaneously, detailed knowledge of the individual parameters is at least mostly of secondary importance. For manufacturers, anomaly detection algorithms with a minimum of parameter settings should be easy to apply and suitable for streaming analysis [4]. Consequently, this type of algorithms should feature mathematical operations with low computational complexity in order to minimize latency in long-running streaming analyses.

**Figure 1:**
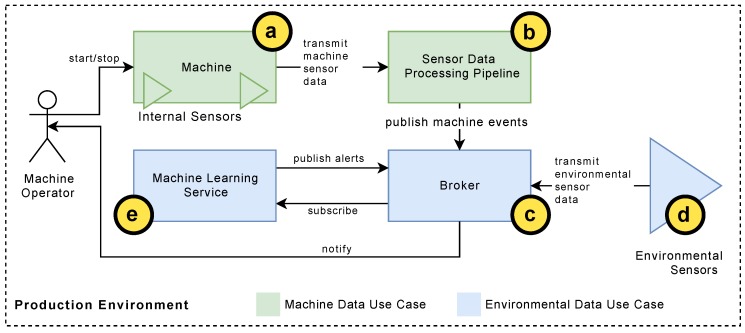
Schematic overview of the presented use cases.

Based on the two real-world examples and their different characteristics to predict anomalies for production machines, this work contributes to the following major insights (see also Figure 1):

- For the pharma packaging machine scenario, sensor streaming data was evaluated with the goal to predict anomalies of one particular machine type. More precisely, machine-internal sensors (see Figure 1a) are acquired and their data is transmitted to a *sensors data processing* service. The latter then evaluates the sensor data with a distance profiling method (see Figure 1b). For this use case, it is shown that the requirement of efficient anomaly detection methods was found, which is also able to cope with the huge amount of sensor data of the analyzed packaging machine. Following this, the pharma packaging company can address other machine types as well.
- For the 3D machine scenario, environmental sensor data was evaluated with the goal to predict anomalies that may affect the production machines. More precisely, temperature sensors (see Figure 1d) send data to a message broker via the Message Queuing Telemetry Transport protocol (MQTT) (see Figure 1c). A *machine learning service* (see Figure 1e) then predicts temperature values that may have negative effects. If a temperature anomaly is detected, the sensor data processing and the machine learning services notify a machine operator via a service message using the message broker. In this use case, several investigated machine learning approaches are presented, which had the goal to efficiently detect anomalies of temperature values. Furthermore, considerations on different sizes of training and test data are discussed.
- For production companies, the detection of anomalies becomes increasingly import. As more research is required to get better insights into real-world scenarios and datasets, this works contributes with the results of two complex use cases. The selection of appropriate algorithms and their parameterization is still challenging, which can be also seen from the fact that less standard software is offered in this context.

The remainder of this paper is organized as follows. First, related works are discussed in Section 2. In Section 3, the method of distance profiling is applied to a real-world dataset of a pharma packaging machine, while Section 4 shows methods to predict anomalies of environmental data of a 3D machine’s manufacturing room. Section 5 discusses the results and deals with the revealed limitations. Section 6 concludes the work with a summary and outlook.

## 2. Related Work

Regarding the **acquisition of sensor data** in manufacturing systems, which is an important prerequisite of this work, different related works exist. The authors of [5] discuss requirements for data acquisition of production systems and introduce an architecture based on the Open Platform Communications Unified Architecture (OPC UA) for data transmission and the precision time protocol (PTP) for time synchronization [6]. The authors of [7], in turn, provided an overview of methods, technologies, and exchange protocols to enable dynamic data acquisition of sensor data in industrial systems. Challenges regarding the representation and transformation of sensor data in cyber-physical systems are presented in [8]. In the field of semiconductor manufacturing, various case studies exist to improve manufacturing processes by analysing manufacturing sensor data [9]. Thus, *Advanced Process Control (APC)* methods utilize control strategies and analyses to identify machine faults and their causes [10]. The results are then used to, for example, optimize maintenance schedules [11,12].

In general, different **anomaly detection techniques** exist, which can be classified into *statistical methods*, such as *Statistical Profiling* [13], *Parametric Statistical Modeling* [14], and *Machine Learning Approaches* [15].

Concerning **use cases of anomaly detection** in industrial systems, the authors of [5] introduce a model-based approach for the prediction of energy consumption in production plants in order to detect anomalies using the ANODA algorithm [16]. [17] detected anomalies by applying a Bayesian network and scoring the resulting features according to a scoring model. The authors of [18], in turn, developed an assistance system for data acquisition, process monitoring, and anomaly detection in industrial and agricultural processes and evaluated three use cases. Anomaly detection was developed individually for each use case and is based, for example, also on the distance profiling approach, with a local outlier factor and PCA-based anomaly detection [19,20].

Regarding **stream-based anomaly detection** in general, different approaches exist. [21] conducted anomaly detection based on an auto-regressive, data-driven model of the considered data stream. They analyzed data streams of real-world wind speed measurements. The authors of [22], in turn, presented an approach based on half-space trees, which is a one-class anomaly detection algorithm. Its advantages with respect to the computational complexity are constant time and memory footprints. Finally, [23] introduced an anomaly detection method based on the Hierarchical Temporal Memory (HTM), which is a stream-based sequence memory algorithm. Furthermore, they introduced datasets containing real-world data streams with labeled anomalies to enable benchmarks for stream-based anomaly detection.

For **time series prediction**, plenty of related works exist. In [24], an algorithm called *Ultra Fast Forest Tree* (UFFT) was investigated that examines the behavior of an ensemble of regression trees on streaming data. The work introduces a hybrid adaptive system for the induction of random forests from streaming data. The UFFT system is an incremental algorithm that poses a constant time complexity to process each instance, works online, and uses the Hoeffding bound to decide when a split test is installed on a leaf leading to a decision node [25]. The algorithm uses a continuous data stream and during the training phase, short-term memory is used. This method, in turn, is restricted to binary classification. However, it can be extended to a multiple classifier by increasing the number of classifiers and building a random forest of binary trees. Although UFFT is not better in prediction, it is significantly faster than the C4.5 algorithm [26]. However, UFFT is not able to detect little or abrupt concept drifts like [27], who investigated drift detection. They present an algorithm, which creates Regression Trees (RTs), instead of Random Forest Trees, based on data streams in the presence of concept drifts. RTs are faster in learning, but are prone to outliers, as they do not work as an ensemble. The *Fast Incremental Regression Tree-Drift Detection* (FIRT-DD) algorithm allows for model adoption at any time and is able to deal with *local* concept drifts and adapts locally. By doing so, global model adoption is avoided to gain efficiency. The change detection algorithm is based on change detection units (CDUs) that monitor the growing process. CDUs require few memory spaces per node and a small, constant amount of time complexity for each sample.

Ref. [27] defined concept drifts as a change of the underlying joint probability, i.e., a change of P(Y|X), and distinguished between three main approaches of concept drifts: (1) methods that explicitly detect concept drifts, (2) methods that use ensembles of decision models, and, (3) methods that are based on data management using a sliding window (similar to our presented approach). *Fast Incremental Model Trees with Drift Detections* (FIMT-DD), the successor of FIRT-DD, is based on randomized model trees and combines them to ensembles, which leads to a random forest regression [28]. Each leaf and node of a tree is built on randomly and independently chosen attributes. The authors additionally created an *Online Regression Forest* (ORF), i.e., out of ten FIMT-DD, with a tree depth of five. However, there is no striking outperformance of one of the presented algorithms when comparing a single online tree, including optional splits with a random forest consisting of ten trees [29]. While performance, in general, depends on the dataset, empirical analyses showed that a single *online option trees with averaging* fits best for most compared datasets.

Altogether, the combination of (1) how anomaly detection methods have been integrated into technical settings of real-world examples of production machines and (2) application examples of how anomaly detection of sensor data can be performed in a data-driven manner, without major parameter optimizations, has not been presented by the discussed other works so far as done in this paper.

## 3. Pharma Packing Use Case—Machine Sensor Data

The following use case presents the application of *distance profiling* to a real-life dataset of a pharma packaging machine with the goal to detect anomalies of a machine component. In the first step, relevant sensor data was collected. Then, the obtained sensor data were processed by a data processing pipeline. Practical results show that distance profiling can be a valuable method to detect the anomalies of the pharma packaging machine components.

**Figure 2:**
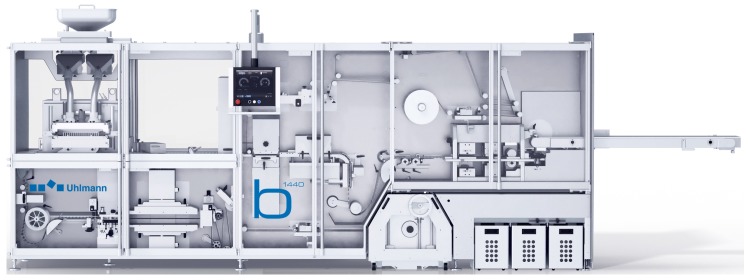
Uhlmann blister machine B1440i.

**Use Case Description:** Uhlmann Pac-Systeme GmbH & Co. KG is a mechanical engineering company headquartered in Laupheim, Germany. Uhlmann is a supplier for pharmaceutical wrapping packaging machines. The blister machines of Uhlmann put tablets into individual packaging units. For example, the offered machines form blisters with individual courts for tablets from a plastic or aluminum foil strand (see Figure 2). Tablets, in turn, are fed and sorted into blister courts. The latter are completed with a cover sheet, while finished blisters are finally punched out by the Uhlmann machines.

As all Uhlmann packaging machines are used in the pharmaceutical industry, country-specific laws and regulations must be considered and fulfilled. These legal requirements are related to the validation of machines, including the provision of detailed documentation of all process steps that are performed during drug packaging. Notably, every packaging machine delivers sensor data, which can be continuously monitored in order to detect anomalies. As an important preliminary technical step for the detection of anomalies, the acquisition of sensor data and the generation of actuator signals must be provided by programmable logic controllers (PLC) [30]. Along with the example of a product loader, this preliminary step will be shortly delineated, as it shows the characterization of the resulting sensor data.

The product loader station of an Uhlmann blister machine loads blisters and leaflets into a carton. The product loader consists of several sensors and servomotors. Specifically, two functional assemblies with servomotors are running in parallel, while the sensors of each single servomotor generate four signals that represent the

1. mechanical position (MP) of the assembly (*ProductLoader.Position*),
2. the difference of a reference value and the actual value of the MP (*ProductLoader.Dref*),
3. the power consumption of the assembly (*ProductLoader.Current*),
4. and the reference signal of the assembly (*ProductLoader.Reference*).

Overall, eight signals per product loader can be acquired. The physical process of product loading and product releasing is executed in continuous cycles. Note that the packaging performance of a machine is therefore expressed in cycles per minute. During these cycles, different anomalies may occur. A machine component can lock, which leads to a production halt. This can be caused, in turn, by a faulty feeding of the packaging box. Furthermore, wear and tear of the ball bearings can lead to increased frictional resistance or a complete failure of the ball bearing lubrication. Both anomalies are reported by the technical evangelists to be detectable by analyzing the power consumption of the product loader.

**Figure 3:**
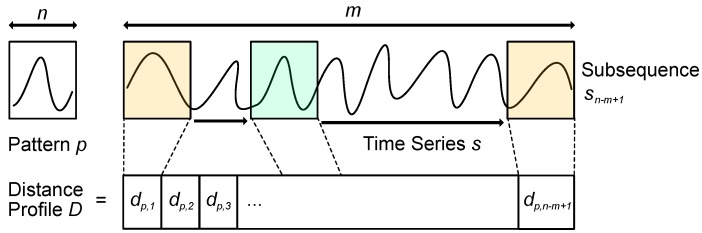
Distance profile for a signal.

**Fundamentals:** To detect anomalies in time series data, distance profiling can be applied [31]. A distance profile, in turn, is a vector *D* of the Euclidean distances between a given time series pattern *p* and every possible subsequence $s_{i}$ in the respective time series *s* (see Figure 3).

In this paper, *Mueen’s Algorithm for Similarity Search (MASS)* was used to calculate the distance profile [32]. When calculating the distance profile, the resulting time complexity is O(nm). MASS uses a convolution-based method to calculate the distance profile of a pattern in O(n×logn). Furthermore, a z-normalization is applied to the generated distance profile vector during the calculation as well. If the Euclidean distance between a pattern *p* and a subsequence $s_{i}$ is smaller than the value of a threshold, then *p* and $s_{i}$ are considered to be similar. **Sensor Data Acquisition:** When processing sensor data of the pharma packing machines, different steps have to be performed [33]. First, sensor data has to be *collected* from a PLC and *transferred* to a collection component. We developed a *binary transmission protocol* to transfer sensor data directly from the PLC to a collection component via TCP sockets. The protocol offers different *frame types* for time synchronization, signal metadata description, and sensor data point transmission.

**Figure 4:**
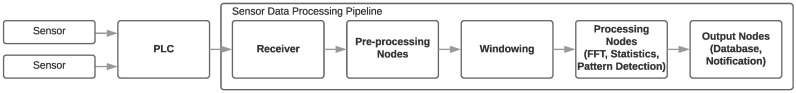
Schema of the sensor data processing pipeline.

**Sensor Data Processing:** The transferred raw data stream has to be split into *windows* and *pre-processed* if it features disturbing noise. After the pre-processing step, the data is *processed* through fast-Fourier transformations and finally *stored* if no further processing steps are required. In this step, we adjusted the already existing technical development to enable the integration of anomaly detection approaches after the pre-processing step. To enable this, the utilized processing pipeline must provide a high variability for every sensor signal, which means that the pipeline is generally difficult to manage and configure. To address these issues, we have developed a *Sensor Data Processing (SDP)* framework in C# of Microsoft. NET for collecting, processing, storing, and visualizing raw sensor data in a continuous processing chain, according to the *data stream processing* model [34]. The SDP defines a graph-based processing model, in which *processing nodes* are connected to each other to handle all aforementioned and required pipeline steps in a controlled way (see Figure 4).

**Figure 5:**
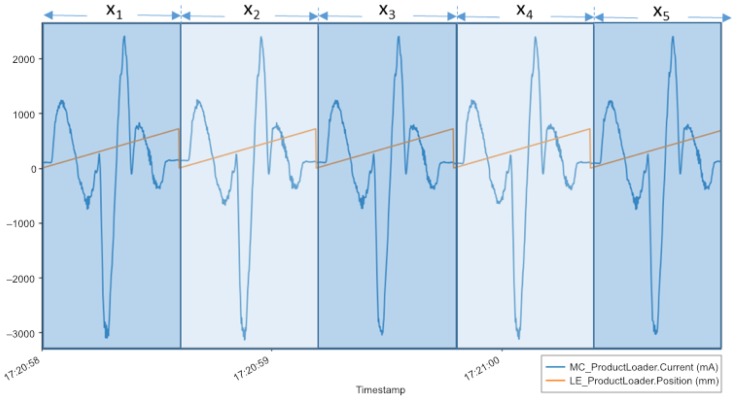
Power consumption (blue) and reference signal (orange) of an assembly.

**Sensor Data Analysis:** The dataset used in this work was generated by an Uhlmann endurance test arrangement in the Uhlmann technical center in Laupheim, Germany. To be more specific, an isolated product loader station was running in a continuous operation mode. For our practical evaluation, sensor data were collected over 10 days by the SDP and stored in a MongoDB database. In total, 1,127,790 timestamps per signal (i.e., time synchronization frames) and 18,736,022,320 data points (i.e., floating-point numbers representing the measured sensor data) were recorded during the mentioned time period, including *NULL* values for the signal gaps. In terms of disk space, the recorded values correspond to roughly 22 GB. Further note that the dataset comprises a few signal gaps due to connectivity losses between the PLC and the storage component. Importantly, the dataset contained records of bearing damage, which eventually led to the failure of the machine. Apart from that, the dataset can be considered as being healthy, as the endurance test and an evaluation of the log data generated by the test execution system showed no other abnormalities. Figure 5 shows the mechanical position and power consumption values of five arbitrary product loader cycles ($x_{1}$ to $x_{5}$) of the considered dataset. During a cycle, the mechanical position values increase monotonously, while a product is loaded into a carton (x-axis). The power consumption (y-axis) shows positive values if the product loader is increasing in its speed, while negative values show breaking actions. When a new cycle starts, the position is set back to zero.

**Distance Profiling:** For this practical use case, the SDP processing pipeline was adjusted by changing the *receiver node* to receive BMTT data, a *windowing node* (see below), and a *processing node* implementing the distance profiling based on the MASS, and an output node to publish MASS results to a broker (see Figure 1c and Figure 4). A *pre-processing node* is not necessary as the *MASS processing node* calculates z-normalization and distance profiles simultaneously. As the SDP pipeline processes data streams, windowing had to be applied [34]. Therefore, *correlation windows* are used. The latter are a specialization of session windows [35]. In contrast to session windows, correlation windows are triggered by one event at the beginning of a new window. In the SDP, correlation windows may be also generated based on other signals. Here, a window trigger is executed based on the master encoder, represented by a saw-tooth signal (see Figure 5). That means, the regular clocking of the master encoder (about 720°) was used to divide the related signals into uniform windows by detecting its falling slope (dataFrame.position[i+1] - dataFrame.position[i]) <−500). The MASS algorithm was then applied to the data of the power consumption signal of the product loader, as it offers the most expressiveness of the mechanical process and features a very high resolution, i.e., one data point per 2 milliseconds. In contrast, the mechanical position offers only about 1000 different, monotonously increasing values (see Figure 5). Moreover, a comparison between the individual patterns becomes possible. Therefore, a new counter value is created for each recognized new pattern of a window. In order to classify a new pattern, the Euclidean Distance is used to determine the signals of existing patterns with the signals of new patterns. In practice, the counter value increases rapidly at the beginning of an analysis series, as well as in the range of any anomaly and drops below a certain limit thereafter. Note that the number of patterns caused by detected anomalies are gradually considered “normal” by MASS, and are therefore smoothed. Conversely, this means that after a large number of detected patterns, it is not assumed that the anomaly has been disappeared.

**Figure 6:**
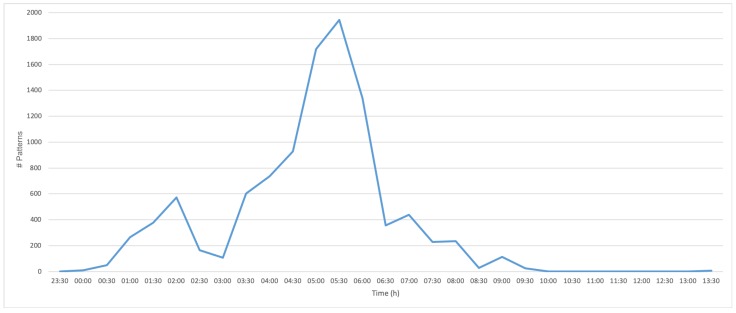
Number of detected patterns (y-Axis) and time (x-Axis) of a product loader of the last 14 h collected.

**Figure 7:**
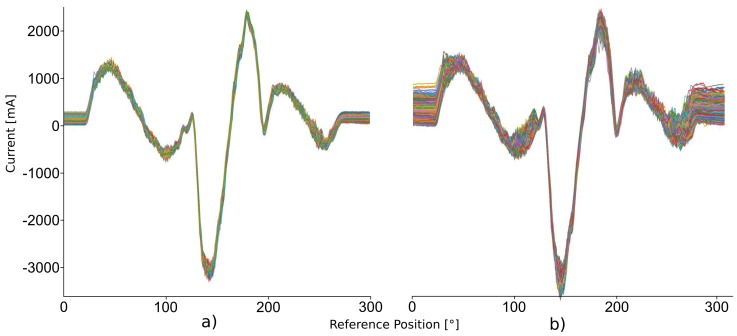
Phase folded plot of (**a**) normal situation with less detected patterns and (**b**) a detected anomaly with many patterns.

**Results:** On the given 10-days dataset, the application of the MASS with the developed SDP processing node took a total run time of 60 min. As a result, the MASS detected fewer than 5 patterns per data point analysis after 22 h in the dataset, i.e., the baseline was reached. Interestingly, about 14 h prior to bearing damage, the number of detected patterns increased rapidly (see Figure 6). At 10:00 a.m., the number of detected patterns returned to 1 pattern per sample. At 01:30 p.m., the number of detected patterns increased again to 7, whereas at 01:48 p.m., the machine finally stopped working due to bearing damage. Figure 7a shows window-layered plots of the power consumption in the normal condition (few detected patterns), whereas Figure 7b in bad condition (many detected patterns). The x-axis represents the reference signal of the product loader in degree, while the y-axis represents the actual power consumption in milliampere (mA) of the two assemblies. In Figure 7b, power consumption shows a higher variance. Interestingly, the idle phase shows the highest variance for power consumption. One reason for this may be the control loop used as during slow movements, the actual position is approached to the reference position with smaller speed changes,. A higher variance also means a lower motor precision of the movements in the normal state.

**Summary:** The dataset of the product loader component of a pharma packaging machine was analyzed to detect anomalies in the collected dataset. The selection of a proper anomaly detection algorithm is challenging as many variants of a machine exist and the selection of parameters for each machine requires high efforts. Therefore, the development of a parameterless algorithm that fits into the setting of the pharma packaging machine should be a major goal. In this paper, a distance profile anomaly detection algorithm was presented and applied. Specifically, the distance profile was calculated with the MASS based on the z-Euclidean Distance. The latter offers various advantages, e.g., it requires few parameters. For the sensor data acquisition, the developed sensor data processing framework (SDP) was used. The latter is based on a processing pipeline model that is configurable and extensible, i.e., other anomaly detection algorithms can be flexibly integrated. Although it is a rather simple method, MASS showed promising results on the presented real-world dataset. It was possible to detect anomalies in a meaningful manner, i.e., based on the number of detected patterns, 13 h as well as 18 min before the damage. Practically, this means that a technical evangelist of the pharma packaging company can better analyze the data to decide whether bearing damage can be avoided by stopping the machine or replacing components before damage actually occurs. However, it has also been shown that the integration of the anomaly detection method requires considerable technical efforts.

## 4. 3D Printing Machine—Temperature Environment Data

The following use case presents the application of prediction models to a real-life dataset of a measuring room of 3D printing machines with the goal to predict anomalies of temperature values. In the first step, relevant sensor data were collected. Then, the obtained sensor data are processed and promising features are selected. Finally, machine learning models predict upcoming values and warn technical machine operators about possible anomalies.

**Use Case Description:** An industrial company in the field of 3D production machines operates a measuring room equipped with nine temperature sensors and additional sensors for *air humidity* (%), *air pressure* (mBar), and *airflow* (m/s). The room contains an arbitrary number of machines and their operators enter and leave the room at arbitrary points in time. Machines within the room are allowed to operate within a temperature threshold, which is defined individually for each machine. A temperature control unit tries to keep the temperature in the room between the upper and lower threshold. The challenge of this project is to figure out temperature anomalies for the measuring room that is solely based on the provided environmental data and does not contain any contextual information of the operated machines, such as the number of operators or the actual number of the machines in the room. Furthermore, no standards for acceptable errors were set for the project. The models presented below return a prediction error on a basis for which the technical evangelists can decide after a notification whether the error rate is acceptable or not.

**Figure 8:**
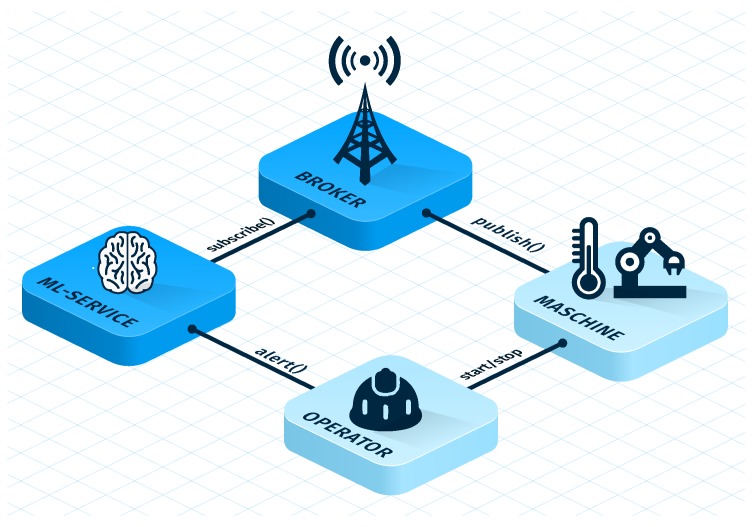
Schematic Architecture of the Environmental Use Case.

**Sensor Data Acquisition:** The measuring room is sending the current sensor information with a transmission rate of one value per 3 min. Figure 8 illustrates the used architecture. Hereby, all values are combined using the *JavaScript Object Notation (JSON)*. Next, all sent messages are transferred via the *Message Queuing Telemetry Transport* protocol (MQTT), which is a publish/subscribe based message protocol for *machine-to-machine* (M2M) communication. The machine learning code is implemented as a separate service using the *scikit-learn python library* [36]. The scikit service subscribes to the environmental values and uses them to train a machine learning model. The model is constantly evaluated and, whenever the predicted temperature value exceeds a defined threshold, the responsible machine operators (i.e., the technical evangelists) are alarmed. In contrast to the first use case, here, the integration of the anomaly detection method is technically easier. However, the selection of proper algorithms is more challenging than for the first use case.

**Figure 9:**
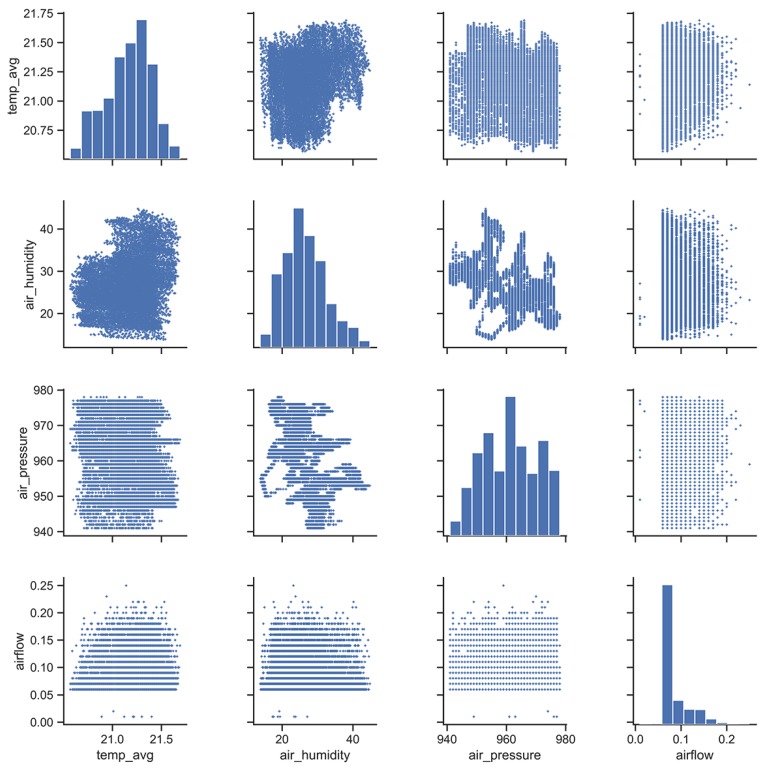
Scatter plots of selected features.

**Sensor Data Processing:** The considered dataset included 115 variables, for which 52 variables contain only *NULL* values. The remaining 63 variables also include *NULL* values, up to 64%. 17,454 rows and 15 columns remain after all *NULL* values have been dropped. As some attributes are directly calculated from others (i.e., change rates), meaning that there is a linear dependency and they have no explainable power to the target, we decided to discard them (see Table 1). Consequently, 13 columns were analyzed. Furthermore, timestamps are used as an index. We created scatter plots of the remaining main variables air_humidity, air_pressure, airflow and temp_avg to enable visual checks for cross-correlations. For example, the scatter plots in Figure 9 are quadratic, meaning that there seems to be no correlation between those three. If there is no correlation between the variables, all of them can be included in the model. Otherwise, the information might be redundant.

If we take a closer look at the nine temperature sensors, we can see that they only vary between 21.99${}^{\left(+\right)}$, as a global maximum, and 20.29${}^{\left(-\right)}$ degrees, as a global minimum. The standard deviation (**std**) varies between 0.20 for temp2 and 0.31 for temp9. The overall mean is 21.27 degrees, which is the mean of temp_avg. We set the *std* in ratio with the mean to calculate the coefficient of variation. The lowest value comes for temp2 and air_pressure, which is 0.009. The highest coefficient of variation is 0.359 for airflow. If we calculate the correlation between temp_avg and the remaining variables, we can obtain that there is a weak correlation between air_humidity and temp_avg (0.22).

**Sensor Data Analysis:** Sensors provide data in an interval of three minutes. As the explanatory variables, we selected humidity, pressure, airflow, and temperature from the past. In the following, a data-driven approach is pursued, since no statement can be made about a *global* cyclicity of the observed data. To train the model for the *prediction*, we need to shift the target temp by 10 rows to predict 30 min of the future. If we want to predict 60 min of the future, we need to shift the target temp by 20 rows. For more or less prediction time periods, the shift works accordingly. We trained three different regressor types, i.e., a *regression tree*, a *random forest*, and a *multi-layer perceptron (MLP)* as a neural network regressor. The advantages of multilayer perceptrons are the capability to learn non-linear models and to learn models in real-time using a partial_fit. The advantages of the regression trees and random forests are their efficiency for the training time and their replicability, eventually enabling meaningful predictions by analyzing the resulting tree structure. When using an **MLP regressor**, too many hidden layers can lead to overfitting. To avoid this, the MLP regressor was gradually supplied with event-driven training data and has been designed with 1 hidden layer containing 100 neurons. For the solver function, we used lbfgs, which is an optimizer in the family of quasi-Newton methods. These models were selected as they are common standard models for which well-documented program libraries, i.e., the Python *scikit-learn* package, exist and whose standard parameterization has been developed by years of community expertise. Recurrent neural networks, for example, are more difficult to train and parameterize and were not considered for these reasons. As we basically address a problem of time series, we did not let the model shuffle the training data and therefore set the shuffle to *FALSE*. For better prediction results with the MLP regressor, it was necessary to scale the data using the StandardScaler implemented in *scikit-learn*. For parameterization, the default values developed by the community were used as a starting point. Therefore, the maximum depth parameter for the **Decision Tree Regressor** was set to 10. Increasing this value leads to overfitting, whereas decreasing the value leads to unsharp results, as the tree structure is not distinct enough. The structure of the **Random Forest Regressor** varied in the tree sizes (between 50 and 1000) and depths (between 5 and 10). As the splitting criterion, we used the *mean squared error*. All parameter settings used can be found in Appendix A.

**Figure 10:**
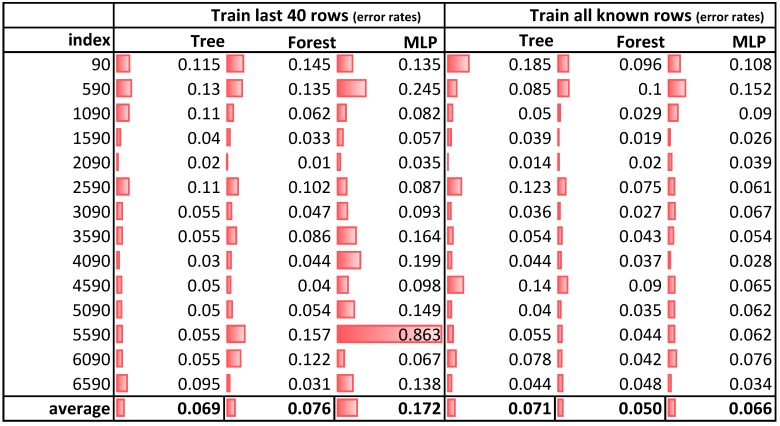
Comparison of the three models based on the two training approaches with a 30 min prediction time.

**Training:** After preparing the data, we split up the dataset into training and test set. Hereby, we used two approaches, for which we compared error rates, based on the three models. In a first approach, we incrementally trained the model with data that have been available so far, thereby the training set grew in each step incrementally with the same size, whereas the size of the testing set stayed fixed at 10 rows, which enabled us for a prediction of 30 min. In a second approach, we also kept the size of the training set fixed for the past 120 min, which corresponds to 40 rows of data and let the model predict the next 30 min. Figure 10 shows error rates for the three models based on data from the past 120 min (“Train last 40 rows”) and for all available data (“Train all known rows”). Thereby, “index” refers to the current row of the training set.

**Figure 11:**
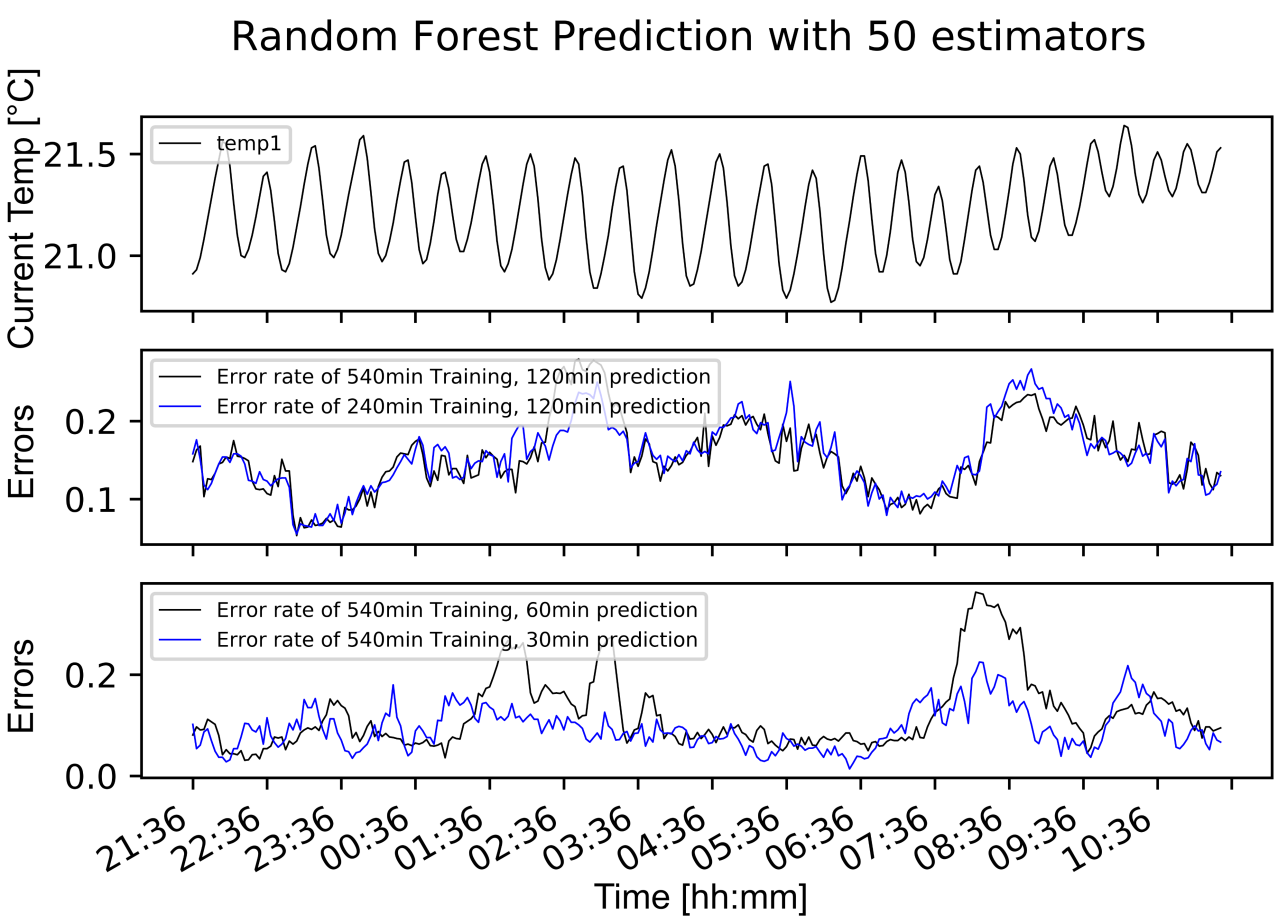
Measured temperature and error rate comparison of Random Forest prediction.

**Figure 12:**
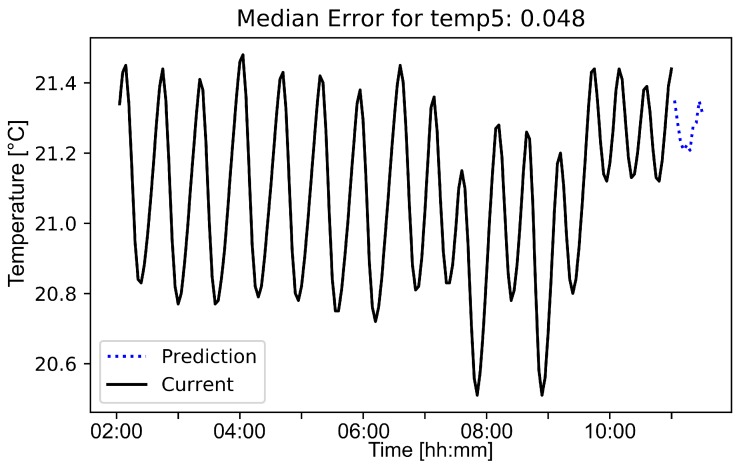
Resulting temperature prediction of half an hour.

**Results:** To compare the error rates over a time period of one day, we visualize errors on different training sizes and different test sizes, as can be obtained from Figure 11. Note that the error rate increases if we increase the prediction time. The amount of training data does not affect the error rates in general, but by decreasing the training time, the variance of the error rate increases. Thus, there is a negative correlation between the variance of the error rate and the training time. In Figure 12, it can be obtained that the random forest approach adapts to the general pattern of the function and is not heavily affected by a changing behavior between 7 a.m. and 9 a.m. Note that *current* in Figure 12 corresponds to the training set, while *prediction* corresponds to the test set. Based on these insights, the technical evangelist can be provided with a useful and efficient method to consider the temperature of the 3D printing machines.

## 5. Discussion

All required data processing steps (acquisition, processing, and analysis) are highly related to the presented use cases. Concerning the acquisition of sensor data, different sensor systems exist. Usually, they use different data models and provide different connection and communication methods (e.g., (a) synchronous communication or message-oriented communication). However, a common exchange model between source and processing system should exist. We briefly summarize the preliminary data processing steps before the anomaly detection methods could be applied. This shows that the anomaly detection requires challenging technical preliminaries that must be considered as well.

In our first use case, we developed a transmission protocol to stream sensor data between one source (PLC) and one sink (SDP framework), while the second use case transfers sensor data from one source to a message broker via MQTT. Using these approaches, the anomaly detection methods could be efficiently integrated. However, some more valuable insights are briefly mentioned. Although for industrial use cases in general, the *Open Platform Communications Unified Architecture (OPC UA)* is widely used to transfer data between systems and to call PLC-related functions [6]. For our use case one, it was not sufficient due to bigger message sizes. Additionally, OPC UA is not designed to continuously transfer large amounts of sensor data. After the acquisition of sensor data, the latter has to be (1) pre-processed in order to convert it into analysis-compliant data models, (2) reduced in its noise as well as (3) being normalized for further analyses. Furthermore, for stream-enabled sensor data, windowing had to be applied for the first use case by creating windows depending on the current machine cycle speed: a faster cycle speed leads to a higher window generation frequency. Thus, windows contain the same physical processes of a machine, which enables their comparison on the other. In general, streaming data architectures may follow the kappa or lambda architecture pattern [37]. The SDP framework follows the kappa architecture, i.e., the collected dataset can be processed by stream replaying. Then, for the second use case, a service collects MQTT messages and evaluates them continuously with the scikit-learn library.

### 5.1. Limitations

For the proposed sensor data analysis using distance profiling, various limitations exist. First of all, the sensitivity of the threshold for the patterns depends on two limiting factors. First, the warm-up phase of the packaging machine must be properly considered, as it does not reflect the normal operation of the machine. For example, lubricants in the considered pharma packaging machine must heat up, which leads to a reduction of mechanical resistance and, thus, to a reduction of power consumption, which, in turn, has to be considered for the anomaly detection (*contextual anomaly*) and the selected thresholds, respectively. Second, there is no common knowledge about a threshold value that indicates a useful alarm setting with respect to the number of detected patterns for a particular machine. Consequently, for the shown results of the distance profiling for the Uhlmann Blister Machine B1440i, it might be the case that our determined threshold can be optimized. Furthermore, pharma packaging machines run with different operating speeds during production that should be considered as well. However, this challenge can be addressed with a *dynamic time warping* [38]. As we analyzed a dataset with a fixed operation speed, this was not necessary for the scenario shown in the work at hand. In order to overcome these limitations, the presented concept can be extended by weighted queries to indicate important sequences within the mechanical insertion process of the product loader.

Concerning the second use case, a major limitation refers to the unknown expected time-series pattern. In contrast to the first use case, the environment of a machine is usually less controlled. For example, we have not considered cases like the opening of a door by a machine operator. As the presented approach is mainly data-driven, it is very vulnerable to effects from sources that are not measured. Furthermore, the number of false alarms, also denoted as false positives, was very high here. The machine operator wants to be informed if a critical state is nearly reached, but the number of false alerts should be minimized to avoid irritations. This aspect is therefore subject to further research.

### 5.2. Summary

Although still many technical limitations came along with the shown results, the insights can provide helpful support for the technical evangelists and machine operators of production companies. On the other, we have shown that preliminary steps are required to integrate the anomaly detection methods into the existing machine settings. Importantly, the integration approaches were accomplished that allow for flexible technical changes. To conclude, the detection of anomalies based on sensor data of a manufacturing machine as well as its environment is useful to support the daily life of machine operators and technical evangelists. On the other, such type of support can be also a starting point to train new machine operators more efficiently.

## 6. Conclusions

In this paper, the collection, processing, and analysis of two real-world data sets from Industry 4.0 scenarios were shown. The first use case focused on sensor data of a pharma packaging machine, for which the data set of the product loader component was analyzed to detect anomalies. Note that the selection of a proper detection algorithm is a challenging task as many machine variants exist and the selection of parameters for each machine requires high efforts. Therefore, a parameterless algorithm had to be developed to support machine operators and technical evangelists. Following this, a distance profile anomaly detection algorithm was presented and applied to the real-life dataset of the product loader. Specifically, the distance profile was calculated with the MASS and based on the z-Euclidean Distance. The latter offers various advantages (e.g., requires only a few parameters).

Furthermore, we showed the analysis and prediction of temperature values in the second use case. Here, the evaluation of different machine learning algorithms revealed promising results. Notably, in contrast to the first approach, the data collection is different as other transmission protocols were used. For example, the frequency of new values was lower in comparison to the first use case, and, therefore, we did not need to use a binary-based transmission protocol, but relied on the standard IoT approach and the MQTT.

Altogether, as production machines become more and more complex, respective companies crave for methods to relieve their technical evangelists in the best possible way. The results of the two uses have shown that this is possible. However, new approaches are still needed to better cope with the variety of settings, sensors, and production machines [39]. Consequently, our future work is driven by the provision of data-driven analytical sensor frameworks in the context of Industry 4.0 production scenarios.

## Figures and Tables

**Table 1:** Data as used for the applied machine learning approach containing 17,454 values. The timestamp is used as an index. Min${}^{\left(-\right)}$ and Max${}^{\left(+\right)}$ values for each single temp variable and are marked in bold for each column.

Variable	Mean	Std	Min	$Q_{0.25}$	$Q_{0.50}$	$Q_{0.75}$	Max
**air_humidity**	26.52	5.94	13.8	22.4	25.8	30.1	44.8
**air_pressure**	961.21	9.11	941	954	962	969	978
**airflow**	0.08	0.03	0.01	0.06	0.07	0.09	0.25
**temp_avg**	21.17	0.23	20.57	21.01	21.2	21.34	21.69
**temp1**	21.23	0.23	20.63${}^{\left(+\right)}$	21.07	21.26	21.4	21.99${}^{\left(+\right)}$
**temp2**	21.26	0.20${}^{\left(-\right)}$	20.61	21.13	21.28${}^{\left(+\right)}$	21.4	21.81
**temp3**	21.11	0.29	20.35	20.92	21.15	21.32	21.89
**temp4**	20.94${}^{\left(-\right)}$	0.26	20.29${}^{\left(-\right)}$	20.77${}^{\left(-\right)}$	20.98${}^{\left(-\right)}$	21.14${}^{\left(-\right)}$	21.55${}^{\left(-\right)}$
**temp5**	21.21	0.23	20.4	21.05	21.24	21.38	21.73
**temp6**	21.18	0.25	20.47	21.01	21.2	21.37	21.82
**temp7**	21.33${}^{\left(+\right)}$	0.22	20.61	21.19${}^{\left(+\right)}$	21.36	21.49${}^{\left(+\right)}$	21.88
**temp8**	21.05	0.24	20.45	20.89	21.08	21.23	21.6
**temp9**	21.10	0.31${}^{\left(+\right)}$	20.31	20.88	21.14	21.34	21.85

## Abbreviations

The following abbreviations are used in this manuscript:

ANODA	ANOmaly Detection Algorithm
FIMT-DD	Fast Incremental Model Trees with Drift Detections
FIRT-DD	Fast Incremental Regression Tree with Drift Detection
JSON	JavaScript Object Notation
HTM	Hierarchical Temporal Memory
IIoT	Industrial Internet of Things
MASS	Mueen’s Algorithm for Similarity Search
MLP	Multi Layer Perceptron
MP	Mechanical Position
MQTT	Message Queue Telemetry Transport Protocol
M2M	Machine-to-Machine
OPC UA	Open Platform Communications Unified Architecture
ORF	Online Regression Forest
PCA	Principal Component Analysis
PLC	Programmable Logic Controller
PTP	Precision Time Protocol
RT	Regression tree
SDP	Sensor Data Processing Framework
UFFT	Ultra Fast Forest Tree

## Appendix A. Parameter Selection for the Evaluation of Environmental Sensor Data

DecisionTreeRegressor( criterion=’mse’,
		max_depth=10,
		max_features=None,
		max_leaf_nodes=None,
		min_impurity_decrease=0.0,
		min_impurity_split=None,
		min_samples_leaf=1,
		min_samples_split=2,
		min_weight_fraction_leaf=0.0,
		presort=False,
		random_state=0,
		splitter=’best’)
		
MLPRegressor( activation=’relu’,
		alpha=0.0001,
		batch_size=’auto’,
		beta_1=0.9,
		beta_2=0.999,
		early_stopping=False,
		epsilon=1e−08,
		hidden_layer_sizes=(100,),
		learning_rate=’constant’,
		learning_rate_init=0.001,
		max_iter=200,
		momentum=0.9,
		n_iter_no_change=10,
		nesterovs_momentum=True,
		power_t=0.5,
		random_state=None,
		shuffle=True,
		solver=’lbfgs’,
		tol=0.0001,
		validation_fraction=0.1,
		verbose=True,
		warm_start=False)

RandomForestRegressor( bootstrap=True,
		criterion=’mse’,
		max_depth=10,
		max_features=’auto’,
		max_leaf_nodes=None,
		min_impurity_decrease=0.0,
		min_impurity_split=None,
		min_samples_leaf=1,
		min_samples_split=2,
		min_weight_fraction_leaf=0.0,
		n_estimators=1000,
		n_jobs=−1,
		oob_score=False,
		random_state=1,
		verbose=0,
		warm_start=False)
